# Correction: Single-cell transcriptome profiling and the use of AID deficient mice reveal that B cell activation combined with antibody class switch recombination and somatic hypermutation do not benefit the control of experimental trypanosomosis

**DOI:** 10.1371/journal.ppat.1012417

**Published:** 2024-07-26

**Authors:** Hang Thi Thu Nguyen, Robin B. Guevarra, Stefan Magez, Magdalena Radwanska

The Acknowledgements section for this article [1] is updated to:

We would like to express our gratitude towards Dr. Joar Esteban Pinto Torres for his support in making our graphical abstract. We also thank members of the Laboratory Animal Research Facility at National Cancer Center, Korea for their technical support related to tissue processing for immunohistochemistry. The authors thank Hien Thi Thu Pham for their contributions to scRNA-seq sample preparation and fine-tuning the research procedure.
