# Supplementary figures and images for: Single-cell transcriptome profiling and the use of AID deficient mice reveal that B cell activation combined with antibody class switch recombination and somatic hypermutation do not benefit the control of experimental trypanosomosis
Source: PLoS Pathog. 2021 Nov 11;17(11):e1010026. doi: 10.1371/journal.ppat.1010026 (PMC8610246; doi:10.1371/journal.ppat.1010026)

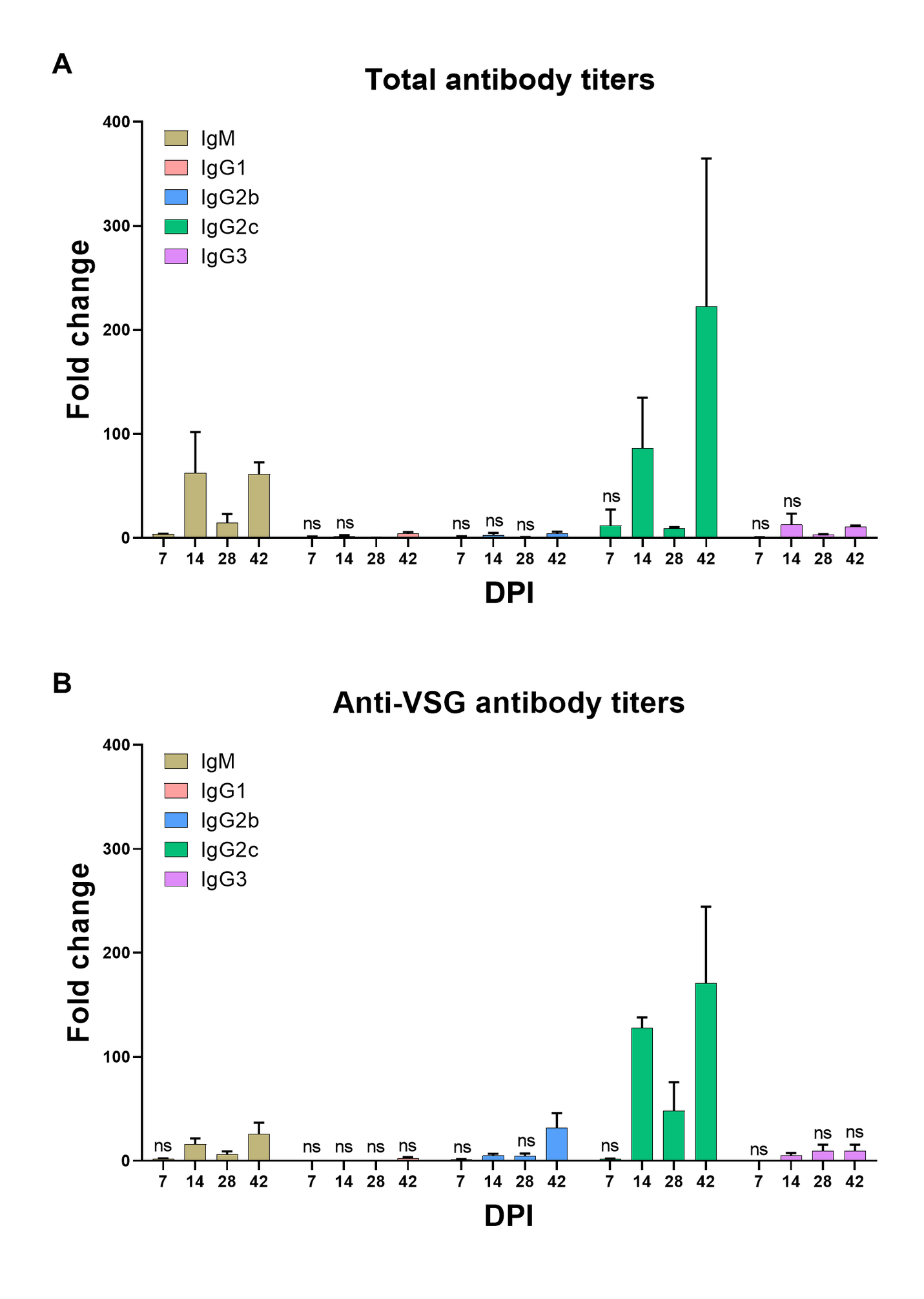

Supplement: S1 Fig — (A) Total IgM, IgG1, IgG2b, IgG2c and IgG3 isotype serum antibody titers during T. evansi infection. (B) Anti-VSG IgM, IgG1, IgG2b, IgG2c and IgG3 isotype serum antibody titers during T. evansi infection. Data is presented as log2 fold change of OD50 values for total antibody titers and End-point titer values for Anti-VSG antibody titers in comparison to Naïve samples. Data is presented as mean + SD of 3 individual samples, from one of three independent experiments, with significant differences compared to naïve control samples ns: non-significant by Student’s t-test. (TIF) [file ppat.1010026.s001.tif]

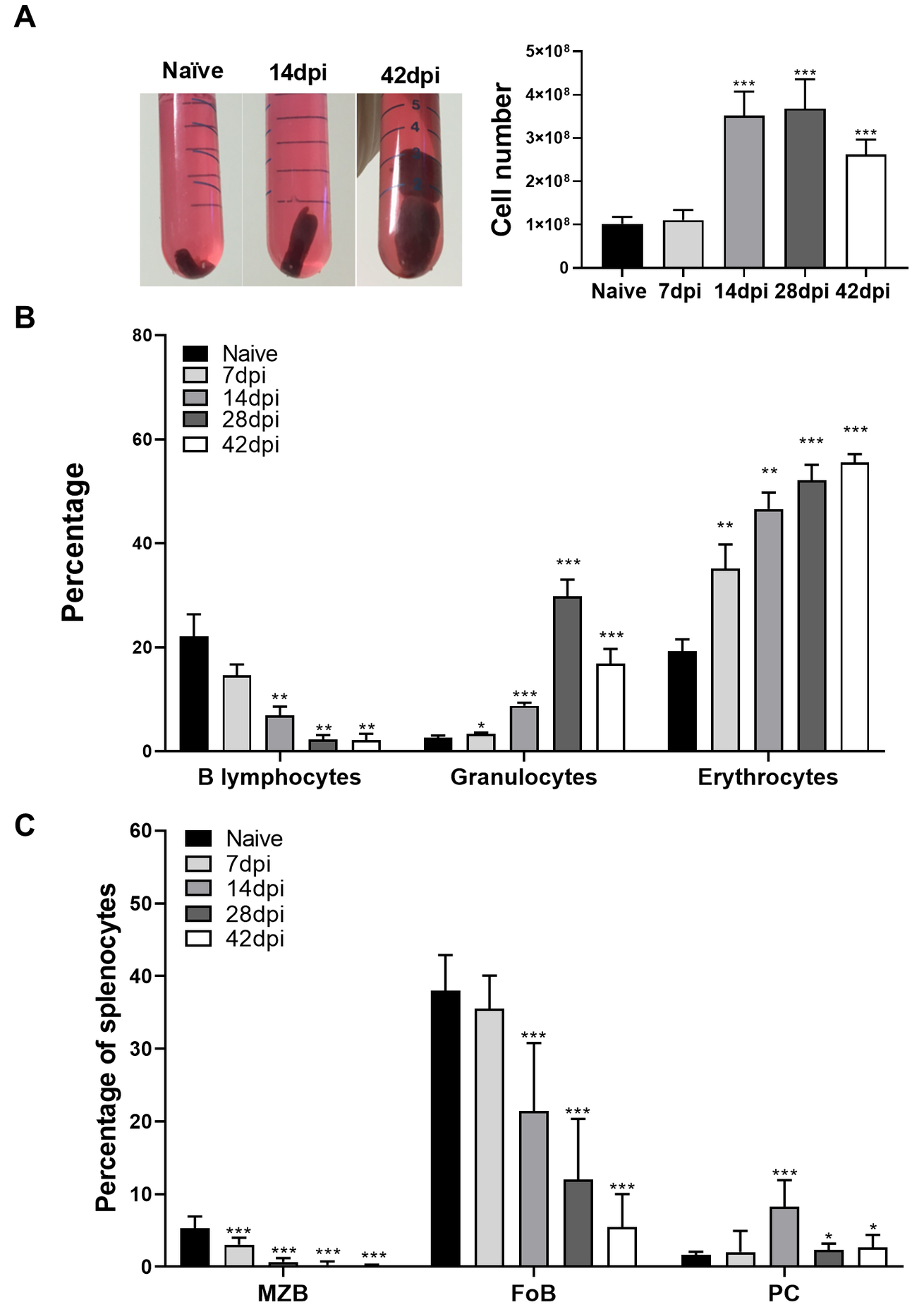

Supplement: S2 Fig — (A) Splenomegaly and splenocytes number at different time points during T. evansi infection. (B) Percentage of B lymphocytes, Granulocytes and Erythrocytes in splenocytes during T. evansi infection. (C) Percentage of B cell populations during T. evansi infection. All data is represented as the mean + SD of three individual samples, in one of three representative experiments, with significant differences compared to naïve control mice * p≤0.05; ** p≤0.01; *** p≤ 0.001by Student’s t-test. (TIF) [file ppat.1010026.s002.tif]

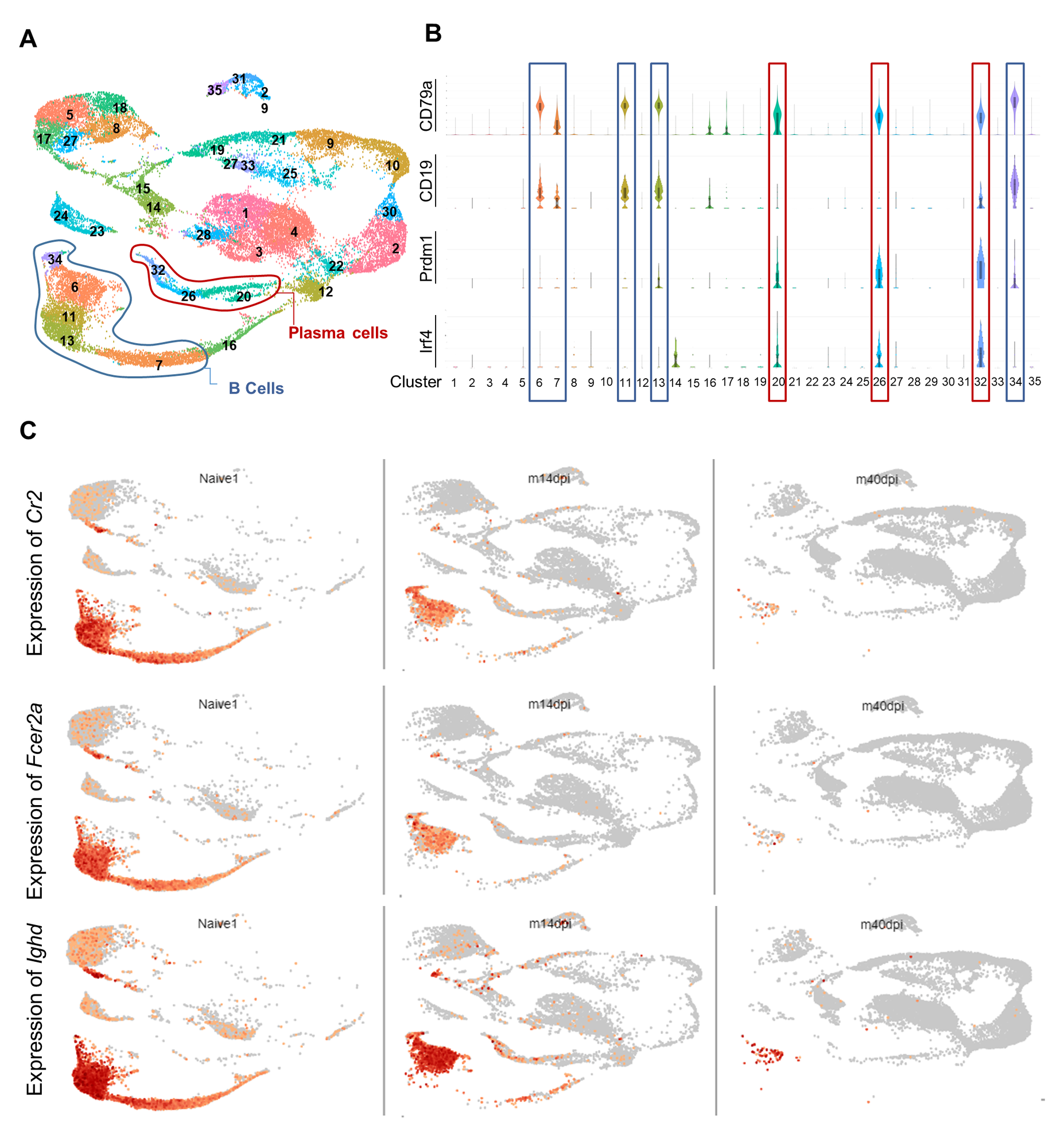

Supplement: S3 Fig — (A) UMAP projection of splenocytes from 3 time points colored by graph-based clusters. (B) Log2 expression of classical markers for B cells (blue boxes) and Plasma cells (Red boxes) across graph-based clusters. (C) UMAP projection of splenocytes colored based on expression of Cr2 (top), Fcer2a (middle), Ighd (bottom). (TIF) [file ppat.1010026.s003.tif]

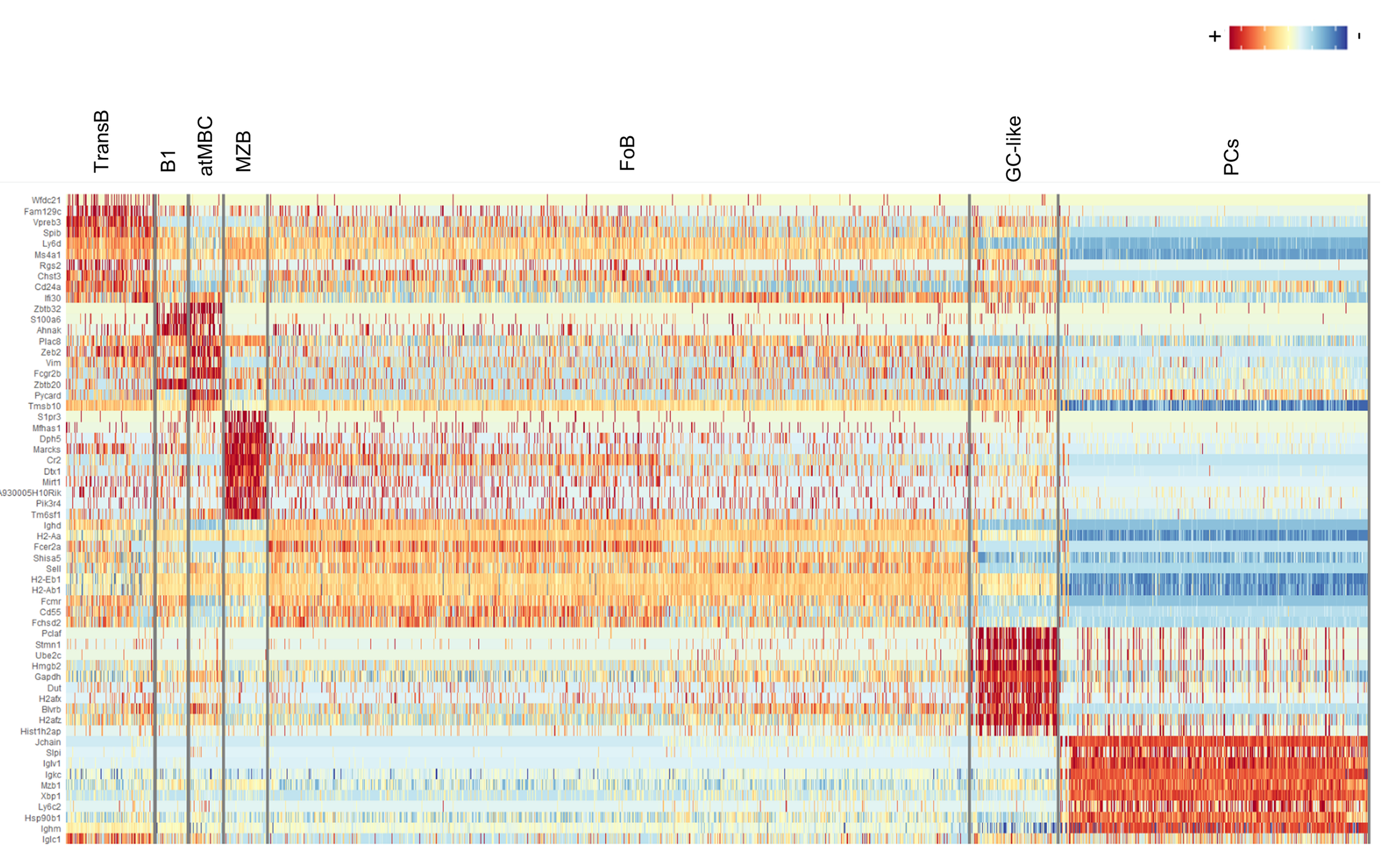

Supplement: S4 Fig — (TIF) [file ppat.1010026.s004.tif]

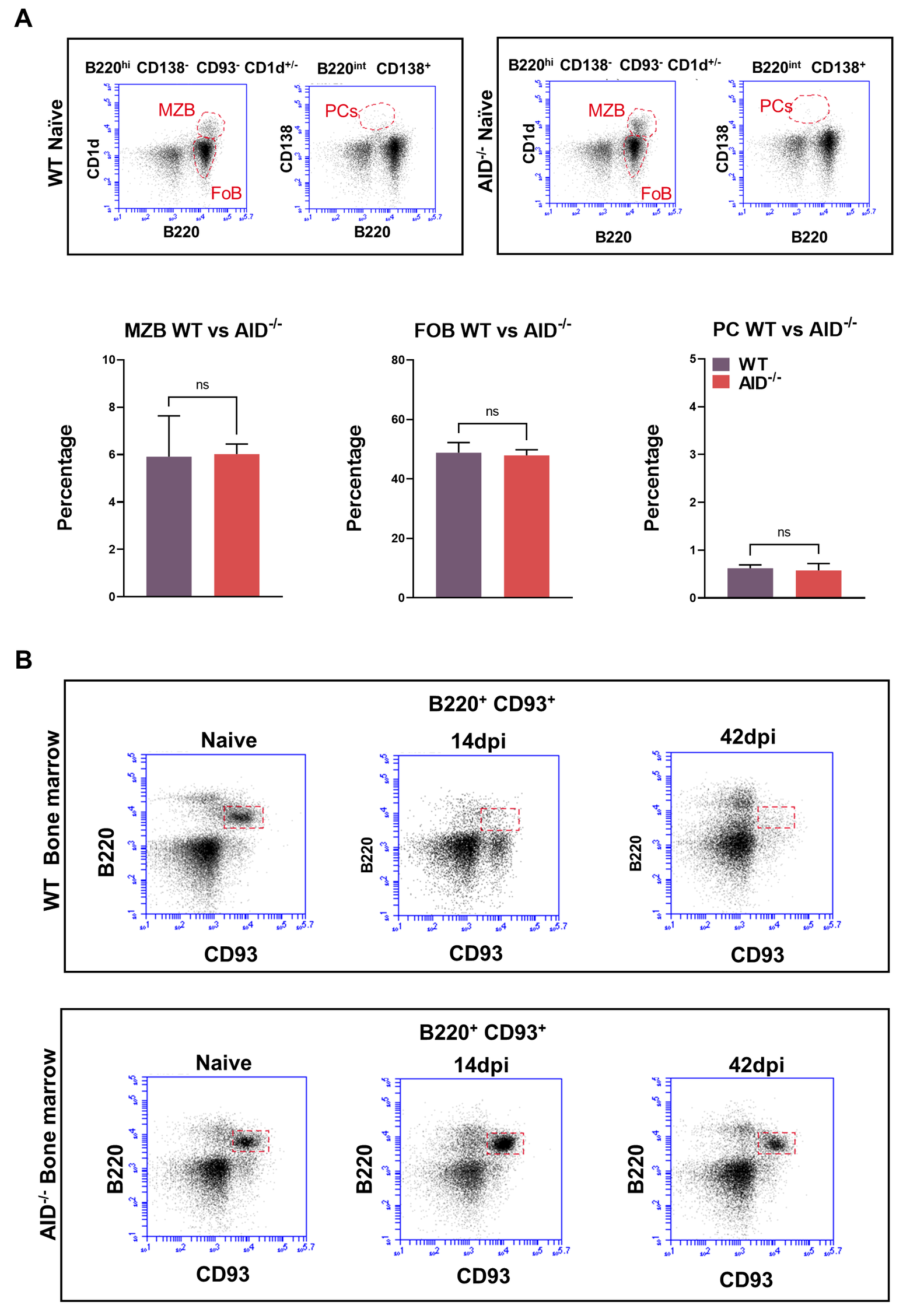

Supplement: S5 Fig — (A) One Representative profile of 9 individual measurements by flow cytometry analysis of MZB, FoB, PCs of WT (upper left) and AID-/- (upper right) naive mice. Percentage values of MZB, FoB and PCs of WT and AID-/- naïve mice (lower panel).) Data is presented as mean + SD, with significant differences compared to naïve WT mice by Student’s t-test. ns: non-significant. (B) Flow cytometry analysis of Early B lineage cells in bone marrow of WT (top) and AID-/- (bottom) infected mice. One representative data set of 9 measurements is shown for each group. (TIF) [file ppat.1010026.s005.tif]

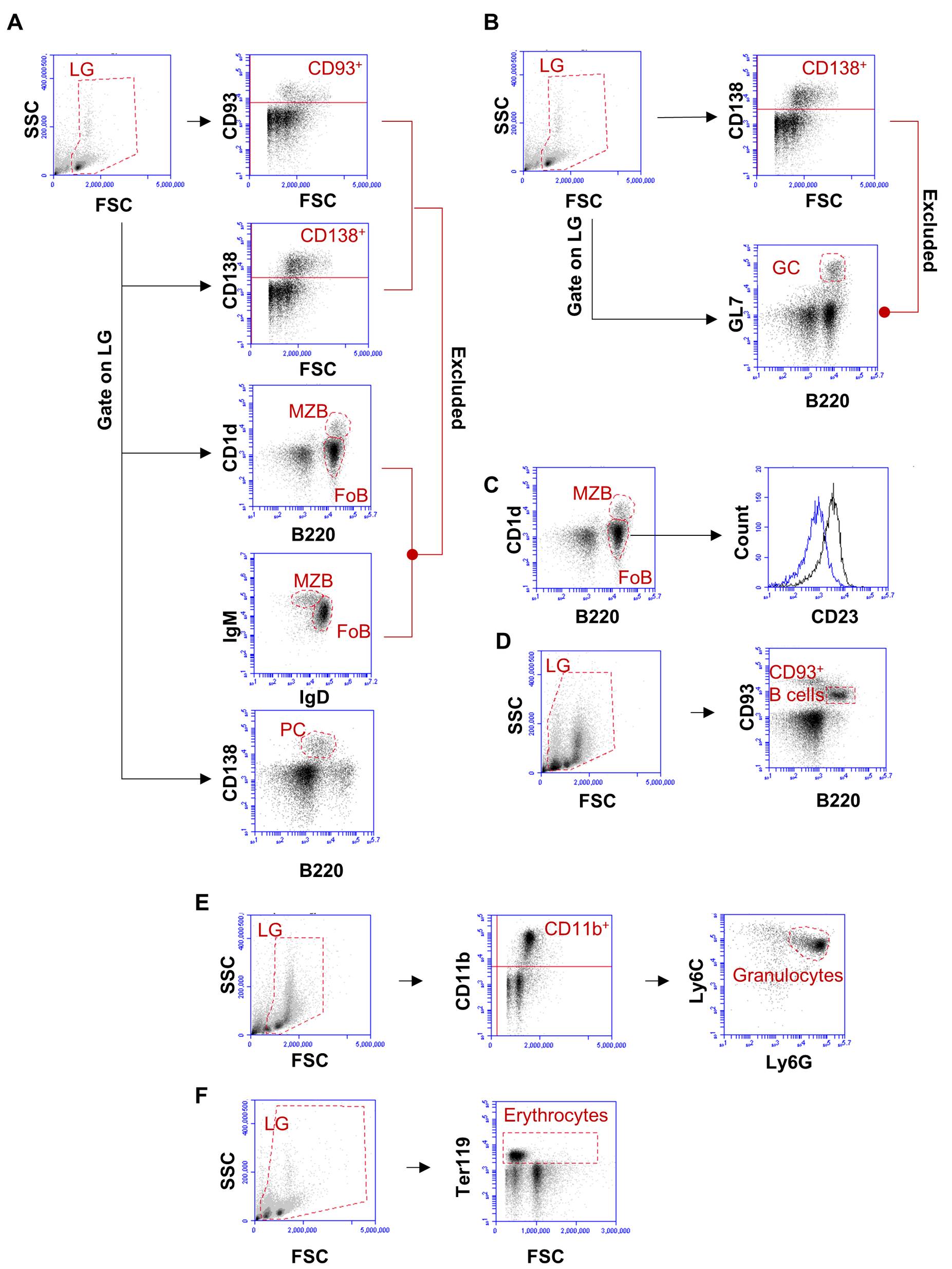

Supplement: S6 Fig — (A-B) Gating strategy to assess cell number and percentage of MZB cells, FoB cells, PCs and GC-like B cells populations. (C) Gating strategy to measure expression of CD23 on FoB cells. (D) Gating strategy to visualize CD93+ early B cell lineage in bone marrow. (E-F) Gating strategy to assess percentage of granulocytes and erythrocytes. (TIF) [file ppat.1010026.s006.tif]
