# Supplementary material for: Single-cell transcriptome profiling and the use of AID deficient mice reveal that B cell activation combined with antibody class switch recombination and somatic hypermutation do not benefit the control of experimental trypanosomosis
Source: PLoS Pathog. 2021 Nov 11;17(11):e1010026. doi: 10.1371/journal.ppat.1010026 (PMC8610246; doi:10.1371/journal.ppat.1010026)
Supplement: S1 Table — (DOCX) [file ppat.1010026.s008.docx]

## **S1 Table: Reagent and resource information**

| REAGENT or RESOURCE | SOURCE | IDENTIFIER |
| --- | --- | --- |
| **Antibodies** | | |
| APC anti-mouse CD23 antibody (Clone B3B4) | BioLegend | Cat# 101620, RRID:AB_2563439 |
| FITC anti-mouse/human CD45R/B220 antibody (Clone RA3-6B2) | BioLegend | Cat# 103206, RRID:AB_312991 |
| APC anti-mouse/human CD45R/B220 antibody (Clone RA3-6B2) | BioLegend | Cat# 103212, RRID:AB_31299 |
| APC anti-mouse CD93 (AA4.1, early B lineage) antibody (Clone AA4.1) | BioLegend | Cat# 136510, RRID:AB_2275868 |
| PE/Cy7 anti-mouse CD93 (AA4.1, early B lineage) antibody (Clone AA4.1) | BioLegend | Cat# 136506, RRID:AB_2044012 |
| PE/Cy7 anti-mouse CD138 (Syndecan-1) antibody (Clone 281-2) | BioLegend | Cat# 142514, RRID:AB_2562198 |
| PE anti-MU/HU GL7 Antigen (T/B Cell Act. Marker) antibody (Clone GL7) | BioLegend | Cat# 144608, RRID:AB_256292 |
| APC anti-mouse IgD antibody ( Clone 11-26c.2a) | BioLegend | Cat# 405714, RRID:AB_10643423 |
| PE anti-mouse TER-119/Erythroid Cells antibody (Clone TER-119) | BioLegend | Cat# 116208, RRID:AB_313709 |
| Alexa Fluor 488 anti-mouse Ly-6G antibody (Clone 1A8) | BioLegend | Cat# 127626, RRID:AB_2561340 |
| PE anti-mouse Ly-6C antibody (Clone HK1.4) | BioLegend | Cat# 128008, RRID:AB_1186132 |
| Purified anti-mouse CD16/32 antibody (Clone 93) | BioLegend | Cat# 101302, RRID:AB_312801 |
| IgM Antibody, PE (Clone X-54) | Miltenyi Biotec | Cat# 130-095-908, RRID:AB_10827707 |
| AID Monoclonal Antibody (mAID-2), eBioscience | Thermo Fisher Scientific | Cat# 14-5959-82, RRID:AB_10669583 |
| Goat Anti-Mouse IgM, Human ads-HRP antibody | SouthernBiotech | Cat# 1020-05, RRID:AB_2794201 |
| Goat Anti-Mouse IgG1, Human ads-HRP antibody | SouthernBiotech | Cat# 1070-05, RRID:AB_2650509 |
| Goat Anti-Mouse IgG2b, Human ads-HRP antibod | SouthernBiotech | Cat# 1090-05, RRID:AB_2794521 |
| Goat Anti-Mouse IgG2c, Human ads-HRP antibody | SouthernBiotech | Cat# 1079-05, RRID:AB_2794466 |
| Goat Anti-Mouse IgG3, Human ads-HRP antibody | SouthernBiotech | Cat# 1100-05, RRID:AB_2794573 |
| Goat Anti-Mouse Ig, Human ads-UNLB antibody | SouthernBiotech | Cat# 1010-01, RRID:AB_2794121 |
| **Chemicals, Peptides, and Recombinant Proteins** | | |
| DMEM High Glucose (4.5 g/l), with L-Glutamine, with Sodium Pyruvate | Capricorn Scientific | DMEM-HPA |
| Fetal Bovine Serum | Atlas Biologicals | Cat#F-0050-A |
| RBC Lysis Buffer (10X) | BioLegend | Cat#420301 |
| BD FACSFlow Sheath Fluid | Biosciences | Cat#342003 |
| Gibco DPBS | Thermo Fisher Scientific | Cat#14190144 |
| ABTS Substrate, Powder | SouthernBiotech | Cat#0202-01 |
| Bovine Serum Albumin lyophilized powder | Sigma-Aldrich | Cat#A2058 |
| Formalin solution, neutral buffered, 10% | Sigma-Aldrich | Cat#HT501128 |
| FSC 22 Frozen Section Media | Leica Biosystems | Cat#3801480 |
| TWEEN20 | Sigma-Aldrich | Cat#P1379 |
| DAPI | Thermo Fisher Scientific | Cat#D1306 |
| Cyanine3 Streptavidin | BioLegend | Cat#405215 |
| Sodium hydroxide | Merck | Cat#1310732 |
| Hypoxanthine | Sigma-Aldrich | Cat#H9636 |
| Bathocuproinedisulfonic acid disodium salt | Sigma-Aldrich | Cat#B1125 |
| 2-Mercaptoethanol | Sigma-Aldrich | Cat#M3146 |
| Thymidine | Sigma-Aldrich | Cat#T9250 |
| L-Cysteine hydrochloride | Sigma-Aldrich | Cat#C7477 |
| Sodium pyruvate | Sigma-Aldrich | Cat#P5280 |
| **Critical Commercial Assays** | | |
| Pierce Coomassie (Bradford) Protein Assay Kit | Thermo Fisher Scientific | Cat#23200 |
| **Experimental Models: Organisms/Strains** | | |
| Mouse: C57BL/6 | Koatech, Korea | N/A |
| Mouse: B6.Cg-Aicda<tm1Hon> (N10)/HonRbrc | Riken Research institute, Japan | RBRC00897 |
| Parasite:*Trypanosoma evansi* Merzouga-93 | Insitute for Tropical Medicine, Antwerp, Belgium | ITMAS150399c |
| **Software and Algorithms** | | |
| Prism9 | GraphPad | N/A |
| C6 Plus Analysis | Biosciences | N/A |
| BioTuring Browser (BBrowser) | BioTurning | N/A |
| R | N/A | N/A |
