# Supplementary material for: Single-cell transcriptome profiling and the use of AID deficient mice reveal that B cell activation combined with antibody class switch recombination and somatic hypermutation do not benefit the control of experimental trypanosomosis
Source: PLoS Pathog. 2021 Nov 11;17(11):e1010026. doi: 10.1371/journal.ppat.1010026 (PMC8610246; doi:10.1371/journal.ppat.1010026)
Supplement: Attachment — Submitted filename: Point-to-point responses.pdf [file ppat.1010026.s010.pdf]

Point-to-point responses:

Reviewer #1: This group has previously shown that infection *of Trypanosoma brucei* induces cell death of splenic B cells and disrupts vaccine-induced memory responses. In the current study, with the use of multiple approaches including flow cytometry and single cell RNA-sequencing analysis, they characterized the antibody and B cell responses in a mouse model of infection with *T. evansi*. They found that the parasites induce activation of splenic IgM^{+}CD1d+ marginal zone and $IgM^{Int}IgD^{+}$ follicular B cells, associated with enhanced plasma levels of IgG2c Abs. They demonstrated that GC-like B cell induction occurs during infection which is independent of GC formation and that ablation of the bone marrow immature B cell compartment prevented mature peripheral B cell replenishment. Finally, they showed that infected $AID^{-/-}$ mice lacking anti-trypanosome IgGs exhibited a better clearance of the parasites compared to infected WT mice with the major contribution of IgM Abs. Overall, this is an insightful manuscript, providing novel insights into our understanding how the parasites escape the host immune responses. I have some minor comments for consideration.

1) In the introduction (line 78-82), the authors mentioned several mechanisms of avoiding killing by the host immune responses including complement. However, recent data demonstrate that complement activation play an essential role in intravascular clearance of trypanosomes.

We do agree with the reviewer that complement-mediated killing is still part of the host's arsenal of tools to control parasitemia, despite adaptations of the parasite to reduce its efficacy. In line 78 the word 'prevents' has been replaced by 'decreases'. The reference of Liu et al has been included as well (In line 434, see below)

2) In the results, Fig. 6, they showed that infected $AID^{-/-}$ mice displayed significantly lower parasitemia. Do infected $AID^{-/-}$ mice survive significantly longer than infected WT mice?

In order to answer this question, we obtained permission for the GUGC IACUC (ethics committee) to repeat three independent experiments with newly imported mice (we have no breeding colony in South Korea available). Results in the Figure 6 (completely reworked figure) show that the improved parasitemia control in the $AID^{-/-}$ mice does not prevent the development of severe infection later-on (post week 3) and also does not result in a prolongation of the lifespan as compared to the fully immune competent mice. The new text (line 254-258) clearly state this observation.

3) In the discussion (line 417-420), they discussed importance of IgM Abs in controlling parasitemia. Consistent with the current study, the authors have previously shown that IgM Abs are required to control the parasitemia during infection with *T. evansi* (Baral et. al., JID 2007 195(10):1513-20; PMID: 17436232), whereas IgM Abs paly a limited role during *T. brucei* infection (Magez et. al. PLoS Pathog. 2008, 4(8):e1000122; PMID: 18688274). **Recent publication demonstrated that IgM Abs play a prominent role in activation of complement for intravascular clearance of *T. congolense* (Liu et. al, 2019 PNAS, 116(48):24214-24220; PMID: 31723045).** As one important part of this manuscript is related to the function of IgM Abs, the author should expand the discussion by comparing the role of IgM Abs among those different trypanosome species. IgM Abs activate complement more efficiently than IgG Abs, given that complement is critically involved in intravascular clearance of trypanosomes. This point should be also considered when comparing IgM and IgG Abs. Finally, in line 419-420: "IgMs are crucial for trypanosome phagocytosis by CR3+ macrophages (100)". This is true for in vitro phagocytosis by purified peritoneal macrophages (ref 100). However, recent in vivo data have shown that complement receptor

CRIg but not CR3 expressed by Kupffer cells plays an essential role in intravascular clearance of *T. congolense* parasites (Liu et. al, 2019 PNAS, 116(48):24214-24220: PMID: 31723045). This knowledge must be updated in the manuscript. Indeed, Kupffer cells express abundant CRIg but limited CR3 in vivo (PMID: 16530040).

We thank the reviewer for pointing out that the Liu et al reference was missing from the first version of the paper, and have included this point in the introduction and discussion, together with a discussion on the superior activity of IgM to activate complement (lines 432-434).

The first point of this comment is of course interesting as well, but it is much harder to discuss. In all honesty, we have the impression that difference between 'T. brucei' and T. evansi could in part be related to an artifact, being that virtually all T. brucei mouse immunology experiments are being conducted with a single cloned parasite (the pleomorphic AnTat 1.1), well adapted to the mouse model through 40+ years of passage. In a way, the same problem applies to T. congolense, where the Tc13 clone has been considered as 'the' model system. While it is easy to understand why these clones have been adopted as 'standard model', there is, in fact no solid data available showing that results obtained with these clones are valid for T. brucei or T. congolense field isolates in general. It would be great if in the future more tools would be available to re-evaluate some of the 'old' data, using non-adapted isolated. In case of T. evansi, the current results obtained with the Merzouga-93 isolate mirror the IgM-dependency for parasitemia control that was published in 2007 with the KETRI2480 stabilate in Baral et. al., JID 2007 195(10):1513-20; PMID: 17436232. We are in the process screening another 10 unrelated T. evansi isolates, as they are available to the research community. However, we are not aware of the existence of such collections for T. brucei/T. congolense at this stage. Hence, to answer the question 'what is the differences between T. brucei and T. evansi' could be…not much, but the T. brucei AnTat 1.1 clone simply does not fully behave anymore like a field isolate, in mice. We have not included this more 'philosophical' answer in the discussion section of this paper, but we are certainly planning on raising the question in the near future, when the comparison between the 10 available T. evansi stabilates is finished.

Part II – Major Issues: Key Experiments Required for Acceptance

Reviewer #1: (No Response)

Part III – Minor Issues: Editorial and Data Presentation Modifications

Reviewer #1: (No Response)

Reviewer #2: This study re-examines a number of issues regarding the role of B cells and antibodies in Trypanosome infection using higher resolution approaches such as single cell RNA sequencing. The authors further provide functional evidence for the role of natural/innate IgM antibody in controlling parasite growth. Their conclusions are that: (1) natural IgM is very important for controlling parasite growth, (2) the "B cell depletion" observed upon infection includes strong reduction in early/transitional B cells (including in bone marrow), (3) germinal center-like B cells are generated, peaking around day 14, even though proper germinal structures don't appear to be present in spleen, (4) innate-like marginal

zone and B1 populations are most strongly depleted, by conversion to short lived effector memory/plasma cells, (5) depletion of follicular B cells is not as complete, and previous studies may have over-estimated this due to CD23 down regulation, (6) IgG responses (particularly IgG2c) are less effective against the parasites than IgM, and may represent an evasion mechanism

Part II – Major Issues: Key Experiments Required for Acceptance

Reviewer #2: This is a very interesting study that clarifies several important aspects, however some conclusions are based on relatively little data and some improvements in data and writing are needed. As the data are mostly observational, the experiments demonstrating functional mechanisms should be strengthened.

1) The data supporting the functional importance of natural IgM *in vitro* is limited. To support the conclusion that IgM is more effective than IgG, isolated IgM and IgG would need to be compared.

We thank the reviewer for this comment and have addressed the concern at several levels. First, we have re-executed all in vitro trypanolysis assays and compared AID$^{-/-}$ mice plasma vs WT mice plasma at 3 different time points: naïve, 7dpi and 14dpi. The results of these assays are displayed in Figure 6C. The results show that both naïve and 7dpi AID$^{-/-}$ mice plasma have a superior lytic activity compared to WT mice plasma. However, to repeat this experiment with isolated IgM and IgGs is not feasible, as AID$^{-/-}$ mice do not have the IgG fraction. In contrast, for WT mice, the superior activity of the IgM fraction has been documented by us earlier in Baral et. al., JID 2007 195(10):1513-20; PMID: 17436232, where it was shown that passive transfer of infection-induced IgMs conferred complete parasite control throughout the experimental period while the IgG fraction did not (Reference 98 – discussion section line 426).

Just for information to the reviewer, the original published data of that experiment are included below.

Figure 4. Parasitemia (A) and survival (B) of mice infected with Trypanosoma evansi and pretreated with infection-elicited purified IgM (black squares) or IgG (white squares) antibodies. Control mice were pretreated with PBS (asterisk) before infection.

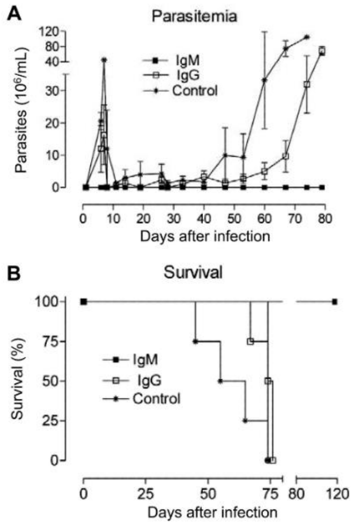

J Infect Dis, Volume 195, Issue 10, 15 May 2007, Pages 1513–1520, https://doi.org/10.1086/515577
The content of this slide may be subject to copyright: please see the slide notes for details.

OXFORD
UNIVERSITY PRESS

Naïve/post-infection serum should be compared. Is AID plasma more effective due to different quantity or quality of IgM Ab or other reasons?

After comparing naïve and post-inoculation plasma (of which the latter is indeed more potent), we addressed the question of quantity versus quality. In fact, in order to make this discussion easier, we have re-plotted the anti-IgM plasma titers of both WT and AID$^{-/-}$ mice represented in figure 6B on one and the same figure, using an x-axis that starts at a 4-times higher dilution for the latter (see below, 1/800 yellow) and the WT mice (grey, stating dilution 1/200). These curves completely overlap, with not a single point where any significant difference between the two lines can be observed. This shows that while AID$^{-/-}$ mice have 4 times higher antibody titers, there is no indication for a better quality of anti-VSG affinity, as the slope of the dilution curves are identical. Finally, while the higher IgM titers can easily be explained by the published phenotype of the AID$^{-/-}$ mice, it would be very hard to think of any biological mechanism that would render these antibodies 'better'. Hence, the answer is that in AID$^{-/-}$ mice, the improved parasitemia control coincides with the higher levels of circulating anti-VSG IgM titers.

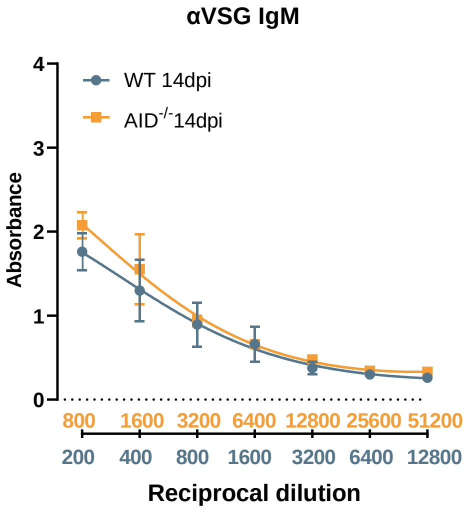

Comment: this new representation of Fig 6B has not been included in the new manuscript. This was only done to have a visual representation of the answer to this question. Both graph lines are however part of the overall Fig. 6B as was the case in the initial document (result description lines 260-263).

2) The data supporting the functional importance of natural IgM *in vivo* is limited. Plasma transfer experiments should compare wild type and AID KO naïve/post-infection serum.

All experiments have been repeated, now including controls and 7dpi data, confirming that plasma from AID$^{-/-}$ mice does have an improved activity when it comes to controlling early stage T. evansi parasitemia.

3) Line 272/73 suggest that IgG2c is "detrimental". Implying that IgG2c is somehow detrimental to the immune response does not seem justified. The data address whether expanded natural IgM is beneficial, but there is not data addressing whether IgG2c is detrimental. To support line 289, where is the evidence that IgG2c is a "decoy mechanism"? This conclusion is repeated several times, and in lines 435 and 460 another mechanism "competition for antigen" is suggested (even though the authors find that the "vast majority of plasma cells are IgM+" on line 385). As there is no evidence in the manuscript for any of these negative effects of IgG2c, these should not be stated as conclusions but as possibilities for future investigation. For this reviewer, the important challenge to focus on is how to boost or maintain IgM responses prior to/during infection, not how to suppress so called detrimental IgG responses.

We fully agree with the reviewer that we have not shown a detrimental impact of IgG2c, and we have tried to remove all suggestions that might lead a reader to conclude this, from the document. We also fully agree with the last statement of the reviewer, that the challenge would be to come up with a strategy to maintain high IgM levels. We hope that the alterations in line 244-248, 449-451 and 457-459 satisfy the reviewer. In line with the comment, we have also slightly adapted title, replacing the word 'undermine' by 'do not benefit'.

Part III – Minor Issues: Editorial and Data Presentation Modifications

Reviewer #2: 1) Line 123. Instead of arbitrarily mentioning day 42, describe the survival curve showing when the mice start to die

Day 42 was chosen for the main late stage flow cytometer and scRNAseq experiments, as we never had a mouse that had succumbed to infection prior to this date in any of the presented or preliminary executed experiments. The survival curve that was present in the first version of the paper has now been described in a different wording (lines 122-123). We have now also clearly indicated the rational for the 42dpi choice for scRNAseq, in lines 140-142.

2) Line 127/28 –5-10 fold increases in IgG isotypes after infection cannot be described as low, just less than IgG2c. To justify the focus of IgG2c, the data comparing anti-VSG titers for different isotypes should be shown.

Following the reviewer's suggestion, we have included data comparing anti-VSG titers for IgM, IgG1, IgG2b, IgG2c and IgG3 together with total antibody titers for those isotypes. The data presented in Figure S1, shows the robust effect on induction of IgM and IgG2c during T. evansi infection. For IgG1, IgG2b and IgG3, the induction was only observed after 4 weeks post injection, and never reached the level of either IgM or IgG2c. The word 'low' has been removed from the text (Line 128).

3) Starting with Fig 1, complete flow cytometry gating strategies need to be illustrated for all cell populations reported.

We have added Figure S6 indicating all gating strategies that we used for our flow cytometry analysis.

4) Line 131 – "decline in cell numbers" Description of flow cytometry data needs to consistently refer to altered cell frequencies, unless referring to absolute cell number data. It's not acceptable to bury absolute cell numbers in supplementary data and not even mention it. I think this should be included in the primary figure and explained clearly as it's critical for the reader to understand what's going on.

In the initial manuscript, we had used cell percentage in Figure 1E to show consistency between all results, especially when comparing flow cytometry and of scRNAseq analysis (of which the latter always refer to portion of cells). However, we fully agree with reviewer that referring cell frequencies as "decline in cell numbers" can causes confusion. To address that issue, we have swapped the cell percentage graph from figure 1E to figure S2C and replaced it with graph showing absolute cell number during infection (previously shown in Figure S2C). We have also adapted our description in main text (lines 129-135).

5) The absolute number data include erythrocytes but method for spleen cell preparation, counting and flow cytometry gating for this analysis are not explained.

We have added cell preparation for erythrocytes staining in our material method part under "Cell isolation and flow cytometry analysis" section (lines 685-687). The gating strategy for estimating the percentage of erythrocytes is included in Figure S6F.

6) Line 141 – what is "graph-based clustering? Phenograph algorithm?

Graph-based clustering is the most popular unbiased clustering algorithm in scRNAseq data analysis which cluster groups of cells based on the similarities of the transcriptomes without any prior knowledge of the labels. In graph-based clustering, vertices are the data points (cells) to be clustered and edges are weighted based on similarity (in transcriptome profile) between data points.

In our analysis, we used a graph-based clustering algorithm which is developed by the Seurat team in Seurat v3 package (Stuart, Butler, et al., Cell 2019). That approach is heavily inspired by PhenoGraph's principle (PhenoGraph, Levine et al., Cell, 2015) in which a K-nearest neighbor graph based on the euclidean distance in principle component analysis space is constructed, and the edge weights between any two cells is refined based on the shared overlap in their local neighborhoods (Jaccard similarity).

7) Line 176 – this concluding statement doesn't make sense as flow cytometry data for several populations have not been shown at this point

We agree with the reviewer, and have removed this sentence.

8) Line 212 - is a relationship between transitional and B1 cell depletion being implied here?

There is no direct relation between the decline in number of splenic Transitional B cells and B1 cells, and therefore we have rephrased the sentence, removing the word 'mirrored' that might have caused confusion (line 213 in the new manuscript version).

9) Line 225/26 - this is a strange way to describe GC B cell expansion – for consistency it would be better to express the result as percentages of total B lineage as in Fig1

We agree with the reviewer that the description in the initial version of the paper could have been confusing and we have re-phrased the sentence. (Line 228)

10) Line 360 "short-lived atMBCs" – what is the evidence they are short-lived?

The term 'short-lived' was not meant here is a <u>de facto</u> reference to the lifespan of these cells (which we have not assessed), but refers to the standard nomenclature used for the population. (Pérez-Mazliah et al. eLife 2018; Knox et al. Immunol Rev. 2019).

11) Line 379 – I don't think histological evidence of absence of GC has been clearly shown. The immunofluorescence images shown do not demonstrate this. Staining such as GL7/IgD/AID would be needed, and comparing infected mice spleen with positive control samples containing GC structures would be needed.

We fully agree with the comment of this reviewer and have rephrased the data description referring to the observations that relate to the destruction of follicular structure, rather than GCs. (Lines 34, 221, 232, 389)

12) Line 405 - how can AID deficiency affect response to IgG-inducing signals? More accurate to say AID-deficient cells can't perform class switch recombination

We agree with the comment of the reviewer and have rewritten the text as suggested (Line 426-427), removing the term 'IgG-inducing signals'.

13) Is the single cell RNAseq data from a single mouse per time point or pooled cells from multiple mice?

We have included in our Materials and Methods, under "Single cell RNA sequencing" section (line 733), that the data was obtained from a single non-pooled sample, in contrast to the flow cytometer were multiple biological (non-pooled) replicates were used to support all the conclusions. (See also comment number 17)

14) Fig 3D - are the gene functional groups shown the most statistically significant ones, or selected based on other criteria?

The ontology analysis results shown in Figure 3D and E, were obtained using the top 100 differential expression genes (Wilcoxon Rank Sum Test) from 14 dpi derived MZB/FoB. The genes were ranked on their fold-change with p-value ≤0.05. This information is mentioned in our Materials and Methods, staring from line 772-774.

15) Fig 4E and F aren't related to A-D parts, thus it's not clear why these should be combined. I felt unclear on the takeaway conclusions regarding B1 cells – unlike MZ cells it seems they are expanded and activated rather than reduced at day 14? This reviewer was not familiar with the markers used to identify the B1 cell population. It would be helpful to compare with flow cytometry analysis of B1 cells as done for all of the other populations.

The reviewer is correct that the minor B cell populations represented in Figure 4 (B1 and atMBC) are not directly related to each other. The reason for combining them in a single figure is related to the very practical fact that we have a limited number of figures that can be included in the paper. Secondly, both populations are 'small' and in fact, the resolution of flow cytometry is not good enough to consistently visualize these populations based on surface markers. However, thanks to the much higher resolution of the scRNAseq approach, these cells could be clustered as separate populations. We think that this is an interesting example of how scRNAseq and flow cytometry can be used in parallel, not just as a confirmation tool, but also as a tool using the uniqueness of both approaches to complement each other. The text in lines 213-215 has been adapted to reflect better this point. All of used gene markers were mentioned with references in Discussion section.

16) Fig 7 should show baseline differences in AID $^{-/-}$

The baseline shown in figure 7B indicates the percentage of cell population in the spleen of the naïve (non-infected) stage, both for WT and AID$^{-/-}$ mice as there is no significant difference between these samples. In the revision, we have added bar graphs comparing percentage of cells between WT naïve mice and AID$^{-/-}$ naïve mice in Figure S5A.

17) In several places the figure legends do not indicate the number of mice. BOTH the number of mice in the experiment shown and the number of repeat experiments should be indicated: Figure 1C (# repeat exp), 1E (how many mice per time point? How many repeats), Fig 2 (how many mice), Fig 4D (# of mice and repeats), Fig 7B (how many repeats), S5B (# of mice, repeats)

All data in bar or line graphs is represented as the mean + SD value of 3 or 5 mice in one of three independent experiment or as mean + SD of 9 values combined. The survival curves represent combined data of three independent experiments. All specific information is added to each figure legend. The scRNAseq experimental results were obtained on one experiment without combining samples, as we are dealing with a non-cloned non-synchronized infection. However, virtually all numerical data obtained in scRNAseq is backed-up by 9 individual flow cytometry observation, mirroring all conclusion and allowing for statistical validation of conclusion with respect to cell population sizes.

18) Numerous typos and wrong words were noted throughout and need to be corrected.

We have tried to correct all errors to the best of our capacity.

Reviewer #3: This is an important manuscript and deserves publication.

There is a long history of work on approaches to a vaccine for trypanosomiasis. Often the work on the pathogen has become divorced form the immunology of the host response. This type of manuscript is well grounded in looking at a realistic animal model (no such models are perfect and some have been shown to be misleading, but this is reasonable) and in using a non-clonal population of a recent field isolate of the parasite.

The approaches used are complementary and produce an interesting result. Importantly, this result may well have general applicability in other pathogen infections such as malaria. The result – that trypanosomes drive an early-stage IgMs/IgG2c switch and terminal PC differentiation followed by reduction of B-cell compartment (little replenishment) – provides a now way of thinking about this infection.

This concept trypanosomes drive immune maturation to evade high avidity IgM interactions and hence subvert the immune response is novel and raises an important concept. Moreover, it is important that the parasite vaccine community is aware of this since focussing on only certain parameters such as general antibody levels etc may well not be good indicators of vaccine effectiveness in the field. Also, it provides a working hypothesis of why bacterial vaccines might fail in tryp infected animals.

This data along with the hypotheses outlined are important and will greatly assist progress of the field. They provide a new set of ideas.

We thank the reviewer for this evaluation, which will strength us to continue this line of research

Part II – Major Issues: Key Experiments Required for Acceptance

Reviewer #3: Overall I have no major points here. The authors use a number of different approaches ...cell analysis, population analysis and markers, plasma transfer, knockout mice to reach a holistic conclusion. I am not an immunologist but in this type of work I think one needs to focus on the holistic rather than always asking for more detail.

We once again thank that the reviewer highlights some important issues here, also relating to the apparent of differences between cloned T. brucei and T. congolense lab strains, and field-related isolates. As mentioned above, these reviewing comments will encourage us to further pursue this line of research.

Part III – Minor Issues: Editorial and Data Presentation Modifications

Reviewer #3: POINTS:

The paper will be of interest to immunologists, parasite immunologists and those interested in the parasite. For the latter the complexities of immunology language use could be clarified in places. For instance in places IgM subtypes are defined but in other places the term “natural IgM” is used. I understand the general meaning of natural IgM and maybe it is just me - but I had a little difficulty

understanding which population of IgM was being referred to …for instance on page 10…the terms Natural IgM, VSG binding IgM, IgMs are used.

We thank the reviewer for this comment and have tried to use this to improve the readability of the manuscript. In the new document we have tried to be as consistent as possible. Textual alterations include in line 43, 58, 302, 414, 416, 449,463,477.

There are a number of English usage / typos that would be usefully amended before publication: for instance particularly in the discussion –

Line 332 treat?

Line 360. lallter?

Line 400 requires result?

Line 417 require to speculate?

Line 427 VSG a homodimer?

Line 431 pulse-chance?

We have tried to correct all errors to the best of our capacity.
