# Supplementary material for: Single-cell transcriptome profiling and the use of AID deficient mice reveal that B cell activation combined with antibody class switch recombination and somatic hypermutation do not benefit the control of experimental trypanosomosis
Source: PLoS Pathog. 2021 Nov 11;17(11):e1010026. doi: 10.1371/journal.ppat.1010026 (PMC8610246; doi:10.1371/journal.ppat.1010026)
Supplement: Attachment — Submitted filename: Comments - PLOS Pathogens-21-00694.docx [file ppat.1010026.s009.docx]

This group has previously shown that infection of *Tryanosoma brucei* induces cell death of splenic B cells and disrupts vaccine-induced memory responses. In the current study, with the use of multiple approaches including flow cytometry and single cell RNA-sequencing analysis, they characterized the antibody and B cell responses in a mouse model of infection with *T. evansi*. They found that the parasites induce activation of splenic IgM^+^CD1d^+^ marginal zone and IgM^Int^IgD^+^ follicular B cells, associated with enhanced plasma levels of IgG2c Abs. They demonstrated that GC-like B cell induction occurs during infection which is independent of GC formation and that ablation of the bone marrow immature B cell compartment prevented mature peripheral B cell replenishment. Finally, they showed that infected AID^-/-^ mice lacking anti-trypanosome IgGs exhibited a better clearance of the parasites compared to infected WT mice with the major contribution of IgM Abs. Overall, this is an insightful manuscript, providing novel insights into our understanding how the parasites escape the host immune responses. I have some minor comments for consideration.

1) In the introduction (line 78-82), the authors mentioned several mechanisms of avoiding killing by the host immune responses including complement. However, recent data demonstrate that complement activation play an essential role in intravascular clearance of trypanosomes.
2) In the results, Fig. 6, they showed that infected AID^-/-^ mice displayed significantly lower parasitemia. Do infected AID^-/-^ mice survive significantly longer than infected WT mice?
3) In the discussion (line 417-420), they discussed importance of IgM Abs in controlling parasitemia. Consistent with the current study, the authors have previously shown that IgM Abs are required to control the prarasitemia during infection with *T. evansi* (Baral et. al., JID 2007 195(10):1513-20; PMID: 17436232), whereas IgM Abs paly a limited role during *T. brucei* infection (Magez et. al. PLoS Pathog. 2008, 4(8):e1000122; PMID: 18688274). Recent publication demonstrated that IgM Abs play a prominent role in activation of complement for intravascular clearance of *T. congolense* (Liu et. al, 2019 PNAS, 116(48):24214-24220; PMID: 31723045). As one important part of this manuscript is related to the function of IgM Abs, the author should expand the discussion by comparing the role of IgM Abs among those different trypanosome species. IgM Abs activate complement more efficiently than IgG Abs, given that complement is critically involved in intravascular clearance of trypanosomes. This point should be also considered when comparing IgM and IgG Abs. Finally, in line 419-420: “IgMs are crucial for trypanosome phagocytosis by CR3^+^ macrophages (100)”. This is true for in vitro phagocytosis by purified peritoneal macrophages (ref 100). However, recent in vivo data have shown that complement receptor CRIg but not CR3 expressed by Kupffer cells plays an essential role in intravascular clearance of *T. congolense* parasites (Liu et. al, 2019 PNAS, 116(48):24214-24220: PMID: 31723045). This knowledge must be updated in the manuscript. Indeed, Kupffer cells express abundant CRIg but limited CR3 in vivo (PMID: 16530040).
